# Antimicrobial resistance of bacteria isolated in a resource-limited region: the experience of the North Kivu Provincial Reference Laboratory in the Democratic Republic of the Congo

**DOI:** 10.3389/fmed.2026.1696339

**Published:** 2026-01-22

**Authors:** Emmanuel Busha Tibasima, Prudence Mitangala Ndeba, Banga Mseza, Ousmane Sy, Stella d’Espérance Assumini Ndeba, Houssein Chalhoub, Raphael Senga, Kasereka Kihemba, Baudouin Byl, Olivier Vandenberg

**Affiliations:** 1 Department of Pediatrics and Child Health, Université Libre des Pays des Grands Lacs, Goma, Democratic Republic of Congo; 2 Université Libre de Bruxelles, Brussels, Belgium; 3 Departement of Epidemiology, Université de Ruwenzori, Butembo, Democratic Republic of Congo; 4 ULB–Coopération, Goma, Democratic Republic of Congo; 5 Department of Pediatrics and Child Health, Faculty of Clinical Medicine and Dentistry, Kampala International University, Ishaka, Uganda; 6 Research and Technology Innovation Unit, Laboratoire Hospitalier Universitaire de Bruxelles-Universitair Laboratorium Brussel (LHUB-ULB), Université Libre de Bruxelles (ULB), Brussels, Belgium; 7 Ami-Labo, Goma, Democratic Republic of Congo; 8 Laboratory Department, Basic Sciences and Pathological Anatomy, Heal Africa Hospital, Goma, Democratic Republic of Congo, Goma, Democratic Republic of Congo; 9 Clinique d’Épidémiologie et Hygiène Hospitalière, Hôpital Erasme, Université Libre de Bruxelles (ULB), Brussels, Belgium; 10 Faculté de Médecine Pharmacie Sciences Biomédicales, Université de Mons, Mons, Belgium; 11 Center for Environmental and Occupational Health, School of Public Health, Université Libre de Bruxelles (ULB), Brussels, Belgium

**Keywords:** antibiotic resistance (ABR), surveillance and mitigating AMR, Democratic Republic of Congo (DRC), Goma, North Kivu, low and middle income countries

## Abstract

**Background:**

Antimicrobial resistance (AMR) is a growing global threat with disproportionate impact in resource-limited settings. We characterized clinically significant bacteria in Goma, Democratic Republic of the Congo (DRC), and their susceptibility using the WHO AWaRe framework.

**Methods:**

We conducted a cross-sectional study (September 2019–March 2022) of routine clinical specimens (blood cultures, urine, vaginal, perineal swabs and pus). Specimens were cultured on standard nonselective (chocolate agar with polyvitamin supplement, fresh blood agar, tryptican broth) and selective media (MacConkey and Chapman agar); isolates were identified locally and referred to the Laboratoire Hospitalier Universitaire de Bruxelles (LHUB-ULB) for confirmation and antimicrobial susceptibility testing (AST).

**Results:**

Overall, 341 isolates underwent AST. *Escherichia coli* was most prevalent (~27%), followed by *Klebsiella pneumoniae* and *Enterococcus faecalis*. Enterobacterales exhibited high non-susceptibility to first- and second-line AWaRe Access agents. In *E. coli*, resistance exceeded 60% to ampicillin, amoxicillin/clavulanate, and ciprofloxacin. *K. pneumoniae* showed uniform resistance to ampicillin and high resistance to cefuroxime, cefotaxime, gentamicin, and colistin. These patterns constrain the effectiveness of commonly used empiric regimens.

**Conclusion:**

AMR is a major public-health problem in Goma. Strengthening laboratory capacity and establishing continuous surveillance are urgent priorities. Recommended actions include participation in WHONET/GLASS program and antibiotic stewardship. In the interim, empiric strategies should favor nitrofurantoin for uncomplicated cystitis, judicious aminoglycoside use where appropriate, early culture, and prompt de-escalation, reserving carbapenems for severe ESBL-risk presentations.

## Introduction

The emergence and spread of multi-resistant bacteria are currently a major public health problem and a veritable health time bomb that is growing in every country in the world (1). Recent data in the literature show worrying results regarding the growing emergence of resistant bacteria producing extended-spectrum beta-lactamases (ESBL), methicillin-resistant strains of *Staphylococcus aureus* or carbapenem-resistant enterobacteria (2).

This worldwide problem involves several intersecting factors linked to the host, the environment and the pathogens. Unfavorable socio-economic conditions such as poverty, inaccessibility to drinking water, poor hygiene, misuse of antibiotics and the illicit sale of counterfeit medicines are all factors contributing to the emergence of multi-resistant bacteria (3, 4). Despite the O’Neill report published in 2016 on significant gaps in monitoring, standard methodology and data sharing as well as recurrent warnings from the World Health Organization (WHO) has addressed the risk of antibiotic resistance, which represents a significant proportion of antimicrobial resistance alongside antiviral and antimalarial (AMR) (5, 6), most Low and Middle Incomes Countries (LMICs) are facing challenges in establishing effective system for surveillance and mitigating AMR (7) and use of the WHO AWaRe system in antibiotic surveillance and stewardship programmes (8). In a study conducted by Craig et al., across African countries, it has been observed that there is a lack of guidelines on antibiotic treatment in compliance with local epidemiology, in all member countries of the African Union (9). Indeed, few studies are available at African level on the catalogues of local or national strains (10).

**Figure 1:**
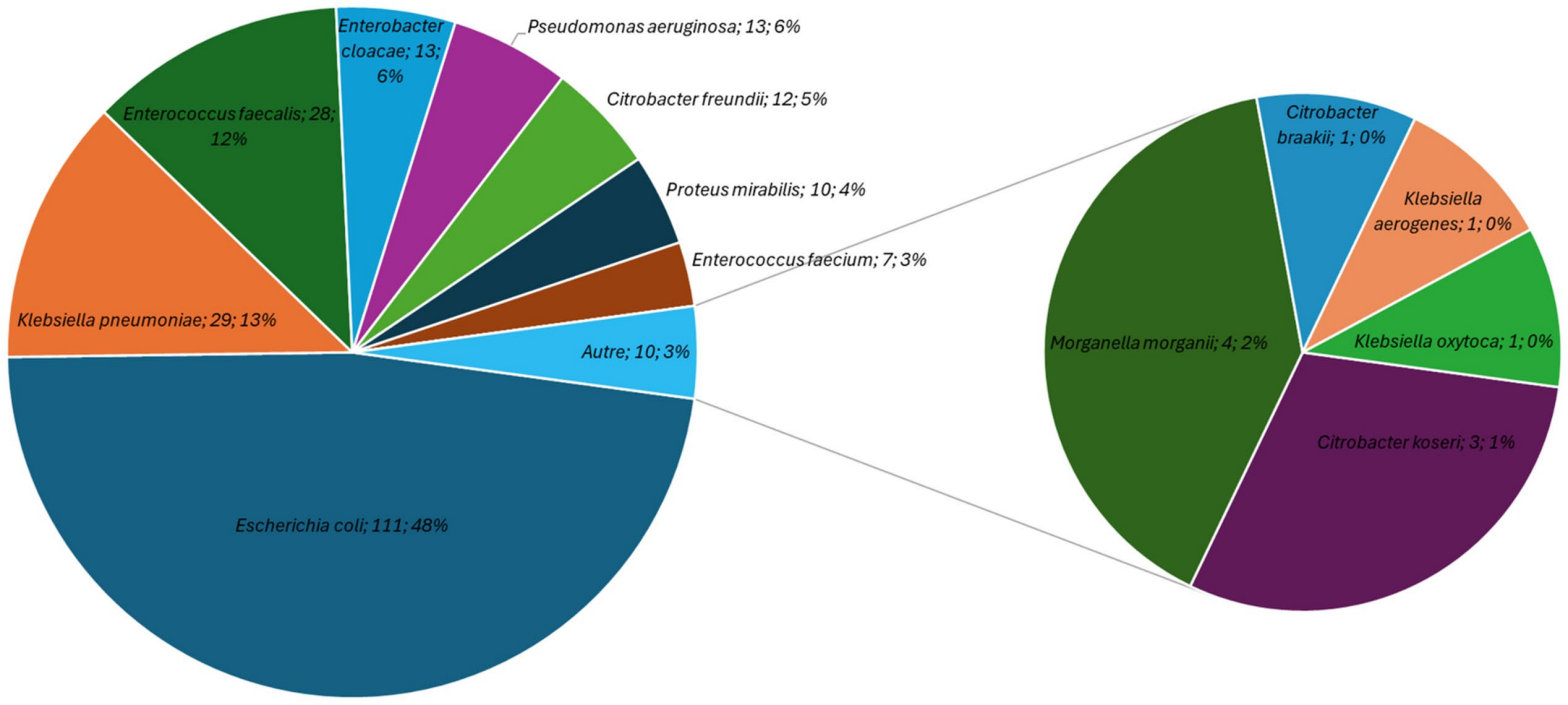
Distribution of bacterial species isolated from various major hospitals in the city of Goma during the study period.

Studies carried out in Democratic Republic of the Congo (DRC), Côte d’Ivoire, Senegal and Kenya on the susceptibility of bacterial pathogens responsible for common respiratory infections to antibiotics show an increasingly high level of resistance (11). Other studies carried out in Uganda (12) Namibia (13) and Tanzania (14) on urinary tract infections also showed an alarming level of bacterial resistance. With the aim of strengthening “antibiotic resistance” (AMR) surveillance and providing the guidance needed to mitigate it, WHO set up the Global Antimicrobial Resistance and Use Surveillance System (GLASS) in 2015 (15) and the WHO AWaRe system in antibiotic surveillance and stewardship programmes in low- and middle-income countries with the goal of achieving an integrated global surveillance system by 2030 to inform and guide local and regional strategies to better contain AMR in particular antibiotic resistance (16) (see Figure 1).

An essential element of this surveillance is improving access to diagnostics, as a microbiology laboratory supports antimicrobial stewardship (AMS) and infection prevention and control (IPC) programs (17). However, laboratory diagnosis is challenging to implement in Low-Resource Settings (LRS) and therefore few hospitals in LRSs are properly equipped with microbiology laboratories because of the limitations of funds and trained staff, with access to diagnostics even more limited at the peripheral (district) level (18). The few studies investigating bacteriological profile and antibiotic susceptibility carried out of the DRC and limited to Bukavu (19), Butembo (20) and Kinshasa (21) do not represent all the specific features of the various regions, given the size of the country.

These studies have mostly show a high level of resistance to antimicrobials, particularly antibiotics, while the health system is weakened by a lack of health infrastructure and by insufficient training and capacity of health personals (21, 22). In addition to the factors described above, the insecurity and war that have plagued eastern DRC for nearly 30 years have not only caused the loss of many lives but have also dismantled the entire local healthcare system increasing the risk of emergence of AMR, given the inadequate use of antibiotics highlighted above but also the destruction of local diagnostic capacities (23).

To our knowledge, the are no studies on the profile of bacteria strains and their sensitivity to antibiotics in the city of Goma. Hence, our choice of blood, urine, vaginal swabs, perineal swabs, and pus samples collected locally in order to better guide the choice of antibiotics.

The aim of our work is to make the scientific community aware of the extent of antibiotic resistance and the diagnostic capacity gap in the city of Goma, located in North Kivu province, one of the most disadvantaged and dangerous regions of the world (24).

## Materials and methods

### Study context

This cross-sectional study was conducted over 3 years between September 2019 and March 2022, to establish a local microbiological profile. The lack of data on bacterial profiles in Goma justifies the choice of this sample. A total of 420 bacterial strains isolated from blood cultures, urine, vaginal swabs, perineal swabs and pus collected as part of routine clinical practice by various hospital laboratories located in the city of Goma (HGR Charité Maternelle, Hôpital Kyeshero, Heal Africa, Hôpital Provincial de Goma, Samaritan Doctors,) were sent to Ami-Labo Provincial Referral Laboratory for further identification. All locally identified bacterial strains were subsequently shipped to the Research and Technology Innovation Unit (RTIU) of the Laboratoire Hospitalier Universitaire de Bruxelles (LHUB-ULB), the reference laboratory for this study to confirm their identification. All strains were bio banked for further analysis.

### Identification

Bacteria were identified locally using standard bacteriologic methods based on Api methods and standard identification gallery according to the availability to reagent and consumables. The various samples received, depending on their type, were steaked using the quadrant method on non specific media (chocolate agar with added polyvitamin supplement, fresh blood agar, tryptican broth) and on specific media (MacConkey and chapman agar). Incubation was performed at 37 °C for 24 to 48 h under a 4% CO2 enriched atmosphere.

For blood cultures, enriched cooked blood agar (Polyvitex) placed under CO2 atmosphere for 48 h or Columbia agar with, 5% blood incubated aerobically for 48 h and anaerobically for 5 days were used. For urine, vaginal and perineal smears, CLED 5 (Cystine Lactose Electrolyte Deficient) agar was used to isolate Gram-negative bacteria. For pus, ANC agar (Nalidixic acid-colistin) or CAP agar (Colistin-Aztreonam) was used to selectively isolate Gram -positive bacteria. The choice of atmosphere (aerobiosis, CO2, or anaerobiosis) depended on the presumptive diagnosis.

All bacterial isolates shipped to RTIU for confirmation of the identification were identified according to Matrix-assisted laser desorption ionization time-of-flight mass spectrometry (MALDI-TOF MS) (MALDI Biotyper, Bruker, Palaiseau, France) (25).

### Antimicrobial susceptibility testing

Due to a lack of reagents resulting from supply insecurity in eastern DRC, no antibiograms were performed in Goma. As a result, the antimicrobial susceptibility tests (AST) presented in this study were performed in Belgium at the reference laboratory using the automated VITEK 2 system (bioMérieux, Marcy-l’Étoile, France) on the most frequently isolated clinically significant isolates. The results were interpreted according to the guidelines European Committee on Antimicrobial Susceptibility Testing (EUCAST, 2013) (26). The detection of ESBL and carbapenemase production detected by the VITEK 2 expert system (27). In addition, antibiotic susceptibility results were presented in this study according to the WHO AWaRe list, which classifies antibiotics into three large groups “Access,” “Watch,” “Reserve” (AWaRe) (28, 29). In which the following antibiotics are classified as belonging to Access group: amoxicillin, ampicillin, amoxicillin/clavulanic acid, sulfamethoxazole/ trimethoprim, cephalothin amikacin, gentamicin clindamycin, cloxacillin, penicillin, nitrofurantoin, oxacillin, tetracycline. The second-line antibiotics on the WHO AWaRe list (fitting in the “Watch” group) are cefotaxime, ceftriaxone, cefuroxime, ciprofloxacin, ceftazidime, piperacillin/tazobactam, vancomycin, erythromycin, norfloxacin and ofloxacin while the “Reserve” antibiotics classified by the WHO AWaRe list are, imipenem, meropenem, ertapenem, colistin (polymyxin E) and fosfomycin.

### Biobanking and shipment of strains

All locally identified isolates were stored in micro-bead vials −80 °C before shipment at −20 °C to the RTIU laboratory following IATA regulation for further studies (30). All cryotubes arriving at RTIU were reseeded on Columbia blood agar 5% sheep blood plates using the direct deposition method before identification and AST.

### Exclusion criteria

Isolates that were non-viable or did not grow, were misidentified, lost during transport were not included. All the bright, large, slightly flattened, yellow colonies on selective Thiosulfate Citrate Bile Sucrose agar (TCBS) after 18 to 24 h of growth at 35 ± 2°C were suspected of being *Vibrio* and were also excluded.

### Statistical analysis

The data received was encoded in an Excel file. They were then cleaned and checked for consistency. The data were analyzed using R Studio 2022.02.3 Build 492.

### Ethics statement

Ethical approval for the study was granted by the Institutional Review Board (IRB) of the Université Libre des Pays des Grands Lacs (ULPGL), (Ref/N°: 001/CE/ULPGL/MK/2019) in accordance with the principles of the Helsinki declaration guaranteeing the rules of confidentiality and respect for data. The study complied with the World Health Organization and international guidelines on antibiotic surveillance for which no recommendation for informed consent has been issued. To ensure confidentiality, samples were analyzed anonymously.

## Results

During the study period, 420 bacterial isolates from 340 human samples (42 blood culture, 51 urine, 131 vaginal swabs, 105 perineal swabs and 11 pus) were identified in AMI-Labo in Goma. All these strains were analyzed locally before shipment to Brussels for further identification. Of the 420 isolates, 90 were excluded from the analysis: 80 were non-viable upon arrival in Belgium, 5 were suspected of being *Vibrio cholerae* and were consequently not sub-cultured in the reference laboratory, 4 were misidentified and 1 was missing from the shipment.

Of the 330 isolates recovered for identification in the reference laboratory, 80 (24.2%) and 250 (75.8%) yielded consistent and inconsistent results, respectively, with their initial identification results obtained in Goma. Because of the low rate of agreement between the identifications performed locally compared with those carried out at reference level, only the results of the analyses performed in the later laboratory are considered here.

Species belonging to the *Enterobacteriaceae* family accounted for 59.4% (196/330) of all identified bacteria, while the *Staphylococcus* or *Enterococcus* genus represented only 11.2% (37/330) of the isolated species. We also observe that non-fermenting bacteria belonging mainly to the genera *Pseudomonas* and *Stenotrophomonas* account for 14.2% (47/330) of the species identified.

Among the 330 isolates, 237 were considered clinically significant isolates whereas 97 were identified as contaminant bacteria and therefore not considered for further analysis. Among the blood culture isolates (18/35) considered as contaminant, we found mostly coagulase-negative *Staphylococcus* spp. (*n* = 5), *Bacillus* spp. (*n* = 5).

Bacteria from the *Enterobacteriaceae* family, along with those of *Pseudomonas* or *Enterococcus genus*, represent the most frequently isolated bacteria in our study on samples from various major hospitals in the city of Goma mainly from urine, vaginal or perineal swabs. Among them, *Escherichia coli* (*n* = 111, 33.6%) was the most frequently identified bacteria followed by *Klebsiella pneumoniae* (*n* = 29, 8.8%), *Enterococcus faecalis* (*n* = 28, 8.5%), *Enterobacter cloacae complex* (*n* = 13, 3.9%), and *Pseudomonas aeruginosa* (*n* = 13, 3.9%) (Figure 1).

These isolates were studied to determine their antimicrobial susceptibility profiles, referring to the antibiotic groups included in the WHO AWaRe list. A summary of the antimicrobial susceptibility profiles of these species based on MIC results is presented in Tables 1–7.

**Table 1:** Susceptibility profile of *Escherichia coli* isolated according to commonly used antibiotics.

Species	AWaRe category	Antibiotic	Class (2)	S	R	I
				*n* (%)	*n* (%)	*n* (%)
*Escherichia coli* (*n* = 111)	Access	Ampicillin	Penicillins	19 (17.12)	92 (82.88)	0 (0.00)
		Amoxiclav^1^	Betalactam/Bêta-lactamase-Inhibitor	35 (31.53)	76 (68.47)	0 (0.00)
		Amikacin	Aminoglycosides	108 (97.30)	2 (1.80)	1
		Gentamycin	Aminoglycosides	88 (79.28)	23 (20.72)	0 (0.00)
		Nitofuratoin	Nitrofuran-derivatives	105 (94.59)	6 (5.41)	0 (0.00)
		TMP/SMX	Sulfonamide-trimetroprim-combinations	22(19.82)	89 (80.18)	0 (0.00)
	Watch	Cefotaxime	Third-generation-cephalosporins	72 (64.86)	39 (35.14)	0 (0.00)
		Cefuroxime	Second-generation-cephalosporins	9 (8.11)	42 (37.84)	60 (54.05)
		Cefuroxime axetil	Second-generation-cephalosporins	71 (63.96)	40 (63.96)	0 (0.00)
		Ciprofloxacin	Fluoroquinolones	32 (28.83)	76 (68.47)	3 (2.70)
		Piperacillin/Tazobactam	Penicillins	99 (89.19)	12 (10.81)	0 (0.00)
		Temocillin	Penicillins	21 (18.92)	1 (0.90)	88 (79.28)
	Reserve	Ceftazidime	Third-generation-cephalosporins	72 (64.86)	34 (30.63)	5 (4.50)
		Fosfomycin	Fosfonics	111 (100.00)	0 (0.00)	0 (0.00)
		Ertapenem	Carbapenems	111 (100.00)	0 (0.00)	0 (0.00)
		Meropenem	Carbapenems	111 (100.00)	0 (0.00)	0 (0.00)

R, Resistant and Intermediate; S, Susceptible.

**Table 2:** Resistance profile of *Klebsiella pneumoniae* isolated according to commonly used antibiotics.

Species	AWaRe category	Antibiotic	Class	S	R	I
				*n* (%)	*n* (%)	*n* (%)
*Klebsiella. pneumoniae* (*n* = 29)	Access	Ampicillin	Penicillins	0 (0.0)	29 (100.0)	0 (100.0)
		Amoxiclav^1^	Betalactam/Beta-lactamase-Inhibitor	17(58.6)	12 (41.4)	0 (0.0)
		Amikacin	Aminoglycosides	28 (96.6)	1 (3.4)	0 (0.0)
		Gentamycin	Aminoglycosides	18 (62.1)	11 (37.9)	0 (0.00)
		Nitofuratoin	Nitrofuran-derivatives	19 (65.5)	10 (34.5)	0 (0.00)
		TMP/SMX	Sulfonamide-trimetroprim-combinations	11 (37.9)	18 (62.1)	0 (0.00)
	Watch	Cefotaxime	Third -generation-cephalosporins	7 (24.1)	22 (75.9)	0 (0.00)
		Cefuroxime	Second-generation-cephalosporins	3 (10.3)	22 (75.9)	4 (13.8)
		Cefuroxime axetil	Second-generation-cephalosporins	7 (24.1)	22 (75.9)	00 (0.00)
		Ciprofloxacin	Fluoroquinolones	11 (37.9)	18 (62.1)	0 (0.00)
		Piperacillin/Tazobactam	Penicillins	22 (75.9)	4 (13.8)	3 (10.3)
		Temocillin	Penicillins	7 (24.1)	0 (0.00)	22 (75.9)
	Reserve	Ceftazidime	Third -generation-cephalosporins	8 (27.6)	20 (69.0)	1 (3.4)
		Fosfomycin	Fosfonics	24 (82.8)	5 (17.2)	0 (0.00)
		Ertapenem	Carbapenems	29 (100.00)	0 (0.00)	0 (0.00)
		Meropenem	Carbapenems	29 (100.00)	0 (0.00)	0 (0.00)

R, Resistant and Intermediate; S, Susceptible.

**Table 3:** Resistance profile of *Enterococcus faecalis* isolated according to commonly used antibiotics.

Species	AWaRe category	Antibiotic	Class	S	R	I
				*n* (%)	*n* (%)	*n* (%)
*Enterococcus faecalis* (*n* = 28)	Access	Ampicillin	Penicillins	28 (100.00)	0 (0.00)	0 (0.00)
		Highly resistant to gentamycin	Aminoglycosides	20 (74.1)	8 (29.6)	0 (0.00)
		TMP/SMX2	Sulfonamide-trimetroprim-combinations	15 (53.6)	13 (46.4)	0 (0.00)
		Nitrofurantoin	Nitrofuran-derivatives	27 (96.43)	1 (3.57)	0 (0.00)
	Watch	Erythromycin	Macrolides	0 (0.00)	28 (100.00)	0 (0.00)
		Clindamycin	Lincosamides	0 (0.00)	28 (100.00)	0 (0.00)
		Imipenem/cilastatin	Carbapenems	7 (25.00)	0 (0.00)	21 (75.00)
		Levofloxacin	Fluoroquinolone	21 (75.00)	7 (25.00)	0 (0.00)
		Moxifloxacin	Fluoroquinolone	21 (75.00)	7 (25.00)	0 (0.00)
		Teicoplanin	Glycopeptides	28 (100.00)	00 (0.00)	0 (0.00)
		Vancomycin	Glycopeptides	28 (100.00)	00 (0.00)	0 (0.00)
	Reserve	Dalfopristin/quinupristin	Streptogramins	1 (3.57)	27 (96.43)	0 (0.00)
		Linezolid	Oxazolidinones	28 (100.00)	00 (0.00)	0 (0.00)
		Tigecycline	Glycylcyclines	28 (100.00)	00 (0.00)	0 (0.00)

R, Resistant and Intermediate; S, Susceptible.

**Table 4:** Resistance profile of *Enterobacter cloacae* isolated according to commonly used antibiotics.

Species	AWaRe category	Antibiotic	Class	S	R	I
				*n* (%)	*n* (%)	*n* (%)
*Enterobacter cloacae* (*n* = 13)	Access	Amoxiclav^1^	Betalactam/Beta-lactamase-Inhibitor	1 (7.7)	12 (92.3)	0 (0.00)
		Amikacin	Aminoglycosides	13 (100.00)	0 (0.00)	0 (0.00)
		Gentamycin	Aminoglycosides	11 (84.61)	2 (15.36)	0 (0.00)
	Watch	Cefepime	Fourth-generation-cephalosporins	9 (69.23)	3 (23.07)	1 (7.69)
		Cefotaxime	Third -generation-cephalosporins	9 (69.23)	4 (30.76)	0 (0.00)
		Cefuroxime	Second-generation-cephalosporins	0 (0.00)	9 (69.23)	4 (30.76)
		Ciprofloxacin	Fluoroquinolones	12 (92.30)	1 (7.69)	0 (0.00)
		Piperacillin/Tazobactam	Penicillins	9 (69.23)	4 (30.76)	0 (0.00)
		Temocillin	Penicillins	2 (15.36)	2 (15.36)	9 (69.23)
	Reserve	Ceftazidime	Third -generation-cephalosporins	8 (61.53)	4 (30.76)	1 (7.69)
		Fosfomycin	Fosfonics	8 (61.53)	55 (38.46)	0 (0.00)
		Ertapenem	Carbapenems	11 (84.61)	2 (15.36)	0 (0.00)
		Meropenem	Carbapenems	13 (100.00)	0 (0.00)	0 (0.00)

R, Resistant and Intermediate; S, Sensitive.

**Table 5:** Resistance profile of *Pseudomonas aeruginosa* isolated according to the routinely used antibiotics.

Germs	AWaRe category	Antibiotic	Class	S	R	I
				*n* (%)	*n* (%)	*n* (%)
*Pseudomonas aeruginosa* (*n* = 13)	Access	Amikacin	Aminoglycosides	5 (38.5)	8 (61.5)	0 (0.00)
		Gentamycin	Aminoglycosides	1 (7.7)	12 (92.3)	0 (0.00)
	Watch	Cefepime	Fourth-generation-cephalosporins	0 (0.00)	11 (84.6)	2 (15.4)
		Cefuroxime axetil	Second-generation-cephalosporins	0 (0.00)	8 (61.5)	5 (38.5)
		Ciprofloxacin	Fluoroquinolones	0 (0.00)	8 (61.5)	5 (38.5)
		Levofloxacin	Fluoroquinolones	0 (0.00)	11 (84.6)	2
		Piperacillin/Tazobactam	Penicillins	0 (0.00)	12 (92.3)	1 (7.7)
		Tircacillin/acide clavulanic	Penicillins	0 (0.00)	12 (92.3)	1 (7.7)
	Reserve	Aztreonam	Monobactam	0 (0.00)	11 (84.6)	2 (15.4)
		Ceftazidime	Third -generation-cephalosporins	0 (0.00)	11 (84.6)	2 (15.4)
		Imipenem	Polymixins	0 (0.00)	8 (61.5)	5 (38.5)

Sensitivity according to EUCAST. R, Resistant and Intermediate; S, Sensitive.

**Table 6:** Resistance profile of *Citrobacter freundii* isolated according to commonly used antibiotics.

Species	AWaRe category	Antibiotic	Class	S	R	I
				*n* (%)	*n* (%)	*n* (%)
*Citrobacter freundii* (*n* = 12)	**Access**	Amoxiclav1	Betalactam/Beta-lactamase-Inhibitor	0 (0.0)	12 (100.0)	0 (0.0)
		Amikacin	Aminoglycosides	12 (100.0)	0 (0.0)	0 (0.0)
		Gentamycin	Aminoglycosides	10 (83.3)	2 (16.7)	0 (0.0)
	Watch	Cefotaxime	Third -generation-cephalosporins	9 (75.0)	3 (25.0)	0 (0.0)
		Cefuroxime	Second-generation-cephalosporins	1 (8.3)	2 (16.7)	9 (75.0)
		Ciprofloxacin	Fluoroquinolones	12 (100.0)	0 (0.0)	0 (0.0)
		Piperacillin/Tazobactam	Penicillins	8 (66.7)	4 (33.3)	0 (0.0)
		Temocillin	Penicillins	2 (16.7)	0 (0.0)	10 (83.3)
	Reserve	Ceftazidime	Third -generation-cephalosporins	9 (75.0)	3 (25.0)	0 (0.0)
		Fosfomycin	Fosfonics	12 (100.0)	0 (0.0)	0 (0.0)
		Ertapenem	Carbapenems	12 (100.0)	0 (0.0)	0 (0.0)
		Meropenem	Carbapenems	12 (100.0)	0 (0.0)	0 (0.0)

R, Resistant and Intermediate; S, Sensitive.

**Table 7:** Resistance profile of *Proteus mirablilis* isolated according to commonly used antibiotics.

Species	AWaRe category	Antibiotic	Class	S	R	I
				*n* (%)	*n* (%)	*n* (%)
*Proteus mirabilis* (*n* = 10)	Access	Ampicillin	Penicillins	0 (0.0)	10 (100.0)	0 (0.0)
		Amoxiclav^1^	Betalactam/Beta-lactamase-Inhibitor	10 (100.0)	1 (10.0)	0 (0.0)
		Amikacin	Aminoglycosides	7 (70.0)	3 (30.0)	0 (0.0)
		Gentamycin	Aminoglycosides	5 (50.0)	5 (50.0)	0 (0.0)
		Nitofuratoin	Nitrofuran-derivatives	0 (0.0)	10 (100.0)	0 (0.0)
		TMP/SMX	Sulfonamide-trimetroprim-combinations	0 (0.0)	10 (100.0)	0 (0.0)
	Watch	Cefotaxime	Third -generation-cephalosporins	0 (0.0)	3 (30.0)	3 (30.0)
		Cefuroxime	Second-generation-cephalosporins	2 (20.0)	3 (30.0)	5 (50.0)
		Cefuroxime axetil	Second-generation-cephalosporins	4 (40.0)	3 (30.0)	3 (30.0)
		Ciprofloxacin	Fluoroquinolones	4 (40.0)	6 (60.0)	0 (0.0)
		Piperacillin/Tazobactam	Penicillins	10 (100.0)	0 (0.0)	0 (0.0)
		Temocillin	Penicillins	2 (20.0)	0 (0.0)	8 (80.0)
	Reserve	Ceftazidime	Third -generation-cephalosporins	6 (60.0)	3 (30.0)	1 (10.0)
		Fosfomycin	Fosfonics	9 (90.0)	1 (10.0)	0 (0.0)
		Ertapenem	Carbapenems	10 (100.0)	0 (0.0)	0 (0.0)
		Meropenem	Carbapenems	10(100.0)	0(0.0)	0(0.0)

R, Resistant and Intermediate; S, Sensitive.

Except for aminoglycosides and nitrofuran derivatives, *E. coli* isolates (*n* = 111) showed resistance above 80% for the tested antibiotics of the access group, whereas among the antibiotics of the watch group, only piperacillin/tazobactam retains activity of 90%. This figure is also observed for antibiotics of the reserve group, except for ceftazidime, for which 35% resistance was observed. In the present study 41.38% (12/29) of *K. pneumoniae* isolates were resistant to amoxicillin/clavulanic acid, and 62.1% (18/29) to sulfamethoxazole/ trimethoprim. All *K. pneumoniae* isolates tested were susceptible to ertapenem or meropenem. *E. faecalis* (*n* = 28) showed 100% resistance to erythromycin and chloramphenicol whereas 52% of isolates were sensitive to sulfamethoxazole/ trimethoprim. Despite the small number of strains tested (*n* = 13), we observe alarming resistance rates for *Pseudomonas aeruginosa* strains which showed resistance to most of the antibiotics tested, including cefepime, ciprofloxacin, levofloxacin, piperacillin/tazobactam, aztreonam, ceftazidime, imipenem (see Tables 5, 8) However, as pointed out by Saegeman et al., the sensitivity results for cefepime and piperacillin/tazobactam should have been checked by a confirmation test using disc diffusion (27).

**Table 8:** Prevalence of ESBL isolates, AmpC phenotype, cephamycin impermeability, cephalosporinase+, carbapenemase.

Species	Total	ESBL	ESBL	Cephalosporinase			Carbapenemase
		ESBL+	ESBL−	AmpC	Acquired Cephalosporinase	Cephalosporinase HN	
*E. coli*	111	83	28	11	8	10	6
*K. pneumoniae*	29	26	3		2	1	1
*P. aeruginosa*	13	2	11			11	10
*E. cloacae*	13	10	3	1		1	
*C. freundii*	12	7	5				
*P. mirabilis*	10	5	5			3	3

Among the 196 *Entobacterales* tested, 74.9% (*n* = 131) were producers of extended-spectrum beta-lactamases, while 86% (*n* = 15) and 5.7% (*n* = 10) produced cephalosporinase and carbapenemase, respectively. *Pseudomonas aeruginosa* showed a high level of multidrug resistance to antibiotics, with 76.9% (*n* = 13) producing carbapenemase (Table 8).

The pie chart illustrates the proportion of isolates attributable to each bacterial species among the clinical samples processed in Goma and confirmed at the University Hospital of Brussels (LHUB-ULB) reference laboratory during the study period, highlighting the relative contribution of the main pathogens identified. *Escherichia coli* was the most frequently identified bacterium, followed *by Klebsiella pneumoniae*, *Enterococcus faecalis/Enterobacter cloacae complex*, and *Pseudomonas aeruginosa*.

## Discussion

In this three-year cross-sectional study from Goma, the clinically significant isolates were dominated by Enterobacterales particularly *Escherichia coli* (*n* = 111; 34%) and *Klebsiella pneumoniae* (*n* = 29; 9%) with additional contributions from *Enterococcus faecalis* (*n* = 28) and *Pseudomonas aeruginosa* (*n* = 13). *Enterobacterales* showed a very high burden of extended-spectrum *β*-lactamase (ESBL) production (75% of tested isolates), while carbapenemase producers were uncommon (6%). *E. coli* exhibited high resistance to several Access-group agents commonly used empirically (ampicillin, amoxicillin/clavulanate, trimethoprim-sulfamethoxazole, and fluoroquinolones), whereas piperacillin/tazobactam retained high activity against *E. coli* in our panel and *K. pneumoniae* remained susceptible to carbapenems in this dataset. *P. aeruginosa* isolates showed multidrug resistance, with a substantial fraction expressing carbapenemase.

Taken together, these findings indicate that empirical regimens commonly used at primary and secondary levels in North Kivu (e.g., aminopenicillins, amoxicillin/clavulanate, and co-trimoxazole) are unlikely to provide reliable coverage for community-onset infections caused by Enterobacterales. The high ESBL burden further reduces the utility of third-generation cephalosporins for empiric therapy of moderate-to-severe infections. Conversely, nitrofurantoin (for lower urinary tract infection) and aminoglycosides (e.g., gentamicin or amikacin, with toxicity monitoring) appear to retain activity against many *E. coli* isolates and remain feasible Access-group options in resource-limited care. For hospital-acquired infections and severe sepsis where ESBL risk is high, early use of a carbapenem may be warranted if available and if the clinical risk is substantial; otherwise, piperacillin/tazobactam may be considered when local susceptibility data support its use, coupled with prompt de-escalation once culture results are available.

A review of studies from eastern Democratic Republic of the Congo (DRC) and neighboring settings shows substantial heterogeneity that likely reflects differences in specimen mix, patient case-mix, care level, and laboratory methods. For example, Bunduki et al. reported from Butembo that *Staphylococcus aureus* (39.8%) was the leading cause of bacteremia, followed by *Listeria* spp. (17.4%), *Moraxella* spp. (14.3%), *Bacillus* spp. (13.4%), and *Klebsiella rhinoscleromatis* (4.1%) (20). This distribution derived from blood cultures differs from ours, which is dominated by Enterobacterales, a discrepancy that is plausibly explained by specimen type (blood vs. predominantly urine and genital/perineal swabs), population differences, and laboratory capacity. In a prospective multicenter study from Gabon, Dikoumba et al. (31) found broadly similar proportions for *E. coli* (34.61%) and *E. faecalis* (9%), but higher frequencies of *K. pneumoniae* (22.63%) and *E. cloacae* (11.91%) than in our series again consistent with variation in clinical settings and sample composition. A retrospective analysis from Rwanda by Habyarimana et al. identified *K. pneumoniae* (31.7%) as the most prevalent isolate, followed by *Acinetobacter* spp. (14.3%), *E. coli* (13.2%), *P. aeruginosa* (2.9%), and *Citrobacter* spp. (0.3%) (32), while Obakiro et al. (12) in Uganda reported *E. coli* (*n* = 442; 33.9%) and *S. aureus* (*n* = 376; 28.8%) as the leading pathogens, with *K. pneumoniae* (*n* = 237; 18.2%) and *S. pneumoniae* (*n* = 76; 5.8%) also represented (11). Collectively, these data suggest that *E. coli* frequently predominates in urinary and some community specimens across the region, whereas the prominence of *K. pneumoniae* and non-fermenters increases in tertiary and hospital-acquired contexts.

In our retrospective observational study, resistance was high among commonly used Access-group antibiotics of the WHO AWaRe classification (28, 29). By contrast, agents categorized as Reserve showed relatively low non-susceptibility likely reflecting restricted availability and use, and thus lower selective pressure. First- and some second-line agents frequently deployed as routine empiric therapy exhibited especially high resistance. Regional molecular studies corroborate these phenotypes: Irenge et al. detected blaCTX-M-15 and blaSHV-18 in *K. pneumoniae* isolates from Bukavu (33), and, in 2019, ST131 was identified in seven *E. coli* urinary isolates from the same city (19). According to GLASS reports (34), resistance exceeding 50% in key pathogens such as *E. coli, K. pneumoniae*, and *P. aeruginosa* is increasingly common globally, constraining the effectiveness of third-generation cephalosporins and, in some settings, driving earlier escalation to carbapenems. These trends reinforce the need to prioritize Access-first empiric choices, obtain cultures rapidly, and practice timely de-escalation.

High resistance rates in our context are consistent with a confluence of factors described across many low- and middle-income countries: non-prescription antibiotic use, variable regulation of dispensing, intermittent medicine quality (including substandard and falsified products), and constraints in diagnostic capacity and quality management (6, 35–38). Market-shaping and policy interventions that ensure dependable supply of high-quality Access antibiotics, while strictly stewarding Watch and Reserve categories, have been proposed as pragmatic levers to slow resistance growth (39–42). At the facility level, our data support:

- Routine, standardized AST with internal quality control and participation in external quality assessment;
- Publication of unit-specific antibiograms to inform purchasing and empiric guidelines;
- Diagnostic stewardship to reduce contamination and improve pre-analytical quality; and
- Prescriber education coupled with audit-and-feedback programs.

### Implications for empirical therapy in North Kivu

For uncomplicated cystitis, nitrofurantoin should be preferred where not contraindicated, and routine empiric use of amoxicillin, amoxicillin/clavulanate, co-trimoxazole, and fluoroquinolones should be avoided given high resistance in *E. coli.* For complicated urinary tract infection/pyelonephritis and other moderate infections likely due to Enterobacterales, an aminoglycoside (e.g., gentamicin or amikacin) can be considered as part of initial therapy where monitoring is feasible, with *β*-lactams adjusted to local susceptibility data and prompt de-escalation on receipt of AST results. For severe sepsis or healthcare-associated infections with high ESBL risk, a carbapenem is appropriate when available and clinically justified; where access is limited, piperacillin/tazobactam may be used if local data indicate activity, recognizing that high ESBL prevalence diminishes the utility of third-generation cephalosporins. Suspected *Enterococcus* infections warrant avoidance of macrolides and chloramphenicol; for lower UTI, nitrofurantoin is preferred when appropriate.

The lack of consistency between the identification results obtained locally and those carried out in the reference laboratory is extremely worrying as it is likely to prevent the development of local treatment guidelines. This discrepancy could be explained by the poor quality of reagents and equipment used locally, the instability of electrical grid, the lack of potable water, the presence of dust and humidity, as well as the level of training and skills of laboratory technicians. All these factors underscore the urgent need to invest in high-performance equipment adapted to local constraints and in the training of laboratory technicians to guarantee the quality and reliability of result (35–37).

### Strengths and limitations

Strengths include a multi-facility sampling frame and reference-laboratory confirmation of organism identification and AST. Limitations include specimen-type skew (predominantly urine and genital/perineal swabs), modest isolate counts for some species, loss of viability during shipment in a subset of locally identified isolates, and limited clinical metadata. Low concordance between local and reference identifications highlights the need for ongoing training, reliable supply chains, and quality management to ensure that laboratory data remain actionable for clinicians.

In conclusion, our study demonstrates that antibiotic resistance has become a major public-health problem in the city of Goma. The pronounced variation in antimicrobial susceptibility underscores the urgent need to strengthen laboratory capacity to enable continuous, high-quality surveillance. Establishing a dedicated mini-laboratory and fostering regional collaboration for antimicrobial-resistance (AMR) surveillance are key, feasible steps toward improved control in resource-limited settings. Building on this, we recommend establishing continuous surveillance platforms (e.g., participation in WHONET/GLASS), expanding sampling to under-represented patient groups and specimen types, and rigorously evaluating the clinical impact of Access-first treatment bundles and antibiotic-stewardship interventions. In the interim, our data support empiric strategies that favor nitrofurantoin for uncomplicated cystitis, judicious use of aminoglycosides where appropriate, routine early culture with rapid de-escalation, and reserving carbapenems for severe ESBL-risk presentations. Collectively, these actions are realistic, high-yield measures to improve patient outcomes and preserve antibiotic effectiveness in North Kivu.

## Data Availability

The raw data supporting the conclusions of this article will be made available by the authors, without undue reservation.
