# Supplementary material for: Antimicrobial resistance of bacteria isolated in a resource-limited region: the experience of the North Kivu Provincial Reference Laboratory in the Democratic Republic of the Congo
Source: Front Med (Lausanne). 2026 Jan 22;13:1696339. doi: 10.3389/fmed.2026.1696339 (PMC12872473; doi:10.3389/fmed.2026.1696339)
Supplement: Supplementary file 2 [file Table_2.DOCX]

**POINT BY POINT RESPONSES**

Dear Reviewers,

Thank you for your invaluable feedback on our manuscript titled " **Antimicrobial resistance of bacteria isolated in a resource-limited region: The experience of the North Kivu Provincial Reference Laboratory in the Democratic Republic of the Congo** " Below, we provide point-by-point responses to address your comments and suggestions.

| **Comments** | **Responses** |
| --- | --- |
| **Reviewer 1: Thank you for your valuable feedback** | |
| **Comment1:**  Abstract:  Method:  The authors mentioned the names of the different facilities involved in their study, but they didn’t mention any method used. The authors are recommended to give a short and clear description of their research methodology rather than listing the names of the labs where they did the research. | Abstract:  We have restructured the summary section while providing answers to various questions.  Method:  Bacterial samples were collected locally from blood cultures, urine, and vaginal and perineal swabs. Depending on their type, the different samples were inoculated onto non-specific media (chocolate agar enriched with multivitamins, fresh blood agar, trypticase broth) and onto specific media (MacConkey agar and Chapman agar).  Due to a shortage of reagents caused by insecurity and supply difficulties in eastern DRC, no antibiograms could be performed in Goma. Therefore, the antimicrobial susceptibility tests (ASTs) presented in this study were carried out in Belgium, at the Technological Research and Innovation Unit of the Reference Laboratory of the University Hospitals of Brussels (LHUB-ULB), using the automated VITEK 2 system (bioMérieux, Marcy-l'Étoile, France). |
| Abstract:  **Comment 2:**  Result:  The authors mention that "341 individuals were identified and subjected to an antibiotic susceptibility test (AST)." "341" what? What identification are the authors referring to? | Abstract:  Result:  341 bacterial strains were identified and subjected to antibiotic susceptibility testing (AST).  "North Kivu Provincial Reference Laboratory, Democratic Republic of Congo." |
| **Comment 3:**  Abstract:  Result: “420 samples, including various cultures (blood, urine, vaginal, and perineal smears), were analyzed at Ami-Labo.” This sentence needs to be removed from the result section. | Abstract:  Result:  The sentence was been removed from the result section |

| **Comment 4:**  Abstract: Result:  The “LHUB-ULB” analysis: what analysis? Be specific. | Abstract:  Result:  The “LHUB-ULB” bacterial strains analysis revelated *Escherichia coli* as the most prevalent bacterium. |
| --- | --- |
| **Comment 5:**  Abstract: The authors are advised to focus on reporting the results clearly and avoid repeating methods used unless it is essential.  The authors report that E. coli and K. pneumoniae exhibited 64.75% resistance to Ampicillin and later claim a 100% resistance to Ampicillin for K. pneumoniae. The authors need to clarify this and uphold consistency. | Abstract: This section has been restructured for clarity (see lines **68 - 74).** |
| **Comment** 6:  Conclusion: The authors are recommended to be specific about what the takeaway from their research is. “Despite limitations”: the authors did not mention any limitations; hence, they are advised to clarify.  “Technologies like the Mini-Lab could provide a solution for monitoring antibiotic resistance”: the authors did not mention this technology in previous sections, and its mention in the conclusion sounds like a big jump with missing steps. | We have revised the conclusion to make it clearer. This restructuring allows for a better understanding of the study's overall idea. We hope it meets your concern. (see line 75-80) |
| **Comment 6:**  Introduction - section:  “In addition, the limited studies that have been carried out in Bukavu (16), Butembo (17), and Kinshasa (18) do not represent all the specific features of the various regions, given the size of the country.” References provided by the authors are unrelated to the locations listed. (16) reports studies done in Eastern Uganda. (17) reports studies done in Namibia, not Butembo. (18) reports studies done in Tanzania, not Kinshasa. The authors are recommended to review their references and cite relevant work properly. | Introduction -section:  We acknowledge the importance of proper referencing in an article. We have checked the references throughout the manuscript **(See line108-114, 495-510).** |

| **Comment 7:**  Introduction - section:  The are plenty of research manuscripts from the DRC published on AMR. Since AMR is a big topic including antibiotics, antivirals, antimalarial drugs, etc., authors are recommended to rewrite their introduction by tailoring it towards the aim of their study, which is “antibiotic resistance” (resistance of bacteria to antibiotics. The authors are free to mention AMR as the big problem and clearly narrow it down to antibiotic resistance. Authors are advised to read more papers on AMR in the DRC and enrich their introduction. | Introduction - section:  We acknowledge that AMR is an important topic encompassing antibiotics, antivirals, and antimalarials. Following your comment, we have rephrased the sentence as follows: The World Health Organization (WHO) has addressed the risk of antibiotic resistance, which represents a significant part of antimicrobial resistance alongside antiviral and antimalarial resistance (AMR). **(See line 98-103)** |
| --- | --- |
| **Reviewer 1: Thank you for your valuable feedback** | |
| **Comment 8:**  Material and methods- section  In the material and methods, the authors wrote the following: “A total of 420 bacterial strains recovered from blood cultures, urine, vaginal swabs, perineal swabs and pus collected”. The authors are advised to include background information in their introduction on antibiotic resistance investigations done using the listed samples and provide their motivation for choosing them. |  |
| **Comment 9:**  Material and methods  “Bacteria were identified locally using standard bacteriologic methods based on Api methods and standard identification gallery according to the availability to reagent and consumables”. The authors are recommended to add a clear description of the methods used. | Material and methods  We have revised the "Materials and Methods" section to include more detailed explanations of the standard bacteriological methods, based on API methods and the standard identification gallery used in this study. We believe these additions will improve understanding of the study's limitations and enhance its quality.  Bacteria were identified locally using standard bacteriologic methods based on Api methods and standard identification gallery according to the availability to reagent and consumables. The various samples received, depending on their type, were steaked using the quadrant method on non specific media (chocolate agar with added polyvitamin supplement, fresh blood agar, tryptican broth) and on specific media (MacConkey and chapman agar). Incubation was performed at 37°C for 24 to 48 hours under a 4 % CO2 enriched atmosphere.  For blood cultures, enriched cooked blood agar (Polyvitex) placed under CO2 atmosphere for 48 hours or Columbia agar with, 5 % blood incubated aerobically for 48 hours and anaerobically for 5 days were used. For urine, vaginal and perineal smears, CLED 5 Cystine Lactose Electrolyte Deficient) agar was used to isolate Gram-negative bacteria. For pus, ANC agar (Nalidixic acid-colistin) or CAP agar (Colistin-Aztreonam) was used to selectively isolate Gram -positive bacteria. The choice of atmosphere (aerobiosis, CO2, or anaerobiosis) depended on the presumptive diagnosis.  All bacterial isolates shipped to RTIU for confirmation of the identification were identified according to Matrix-assisted laser desorption ionization time-of-flight mass spectrometry (MALDI-TOF MS) (MALDI Biotyper, Bruker, Palaiseau, France) **(See lines 158-174 ).** |
| **Comment 10**:  Material and methods  “A total of 420 bacterial strains recovered from blood cultures, urine, vaginal swabs, perineal swabs and pus collected from a routine clinical perspective in various hospital laboratories located in the city of Goma were sent to Ami-Labo Provincial Referral Laboratory for further identification.” The authors are recommended to provide the number of the labs where samples were collected from, as well as their names and locations. | We have incorporated the hospital laboratories from which the samples originated  “A total of 420 bacterial strains isolated from blood cultures, urine, vaginal swabs, perineal swabs and pus collected as part of routine clinical practice by various hospital laboratories located in the city of Goma (**HGR Charité Maternelle, Hôpital Kyeshero, Heal Africa,** **Hôpital Provincial de Goma, Samaritan Doctors)** were sent to Ami-Labo Provincial Referral Laboratory for further identification. All locally identified bacterial strains were subsequently shipped to the Research and Technology Innovation Unit (RTIU) of the Laboratoire Hospitalier Universitaire de Bruxelles (LHUB-ULB), the reference laboratory for this study to confirm their identification. All strains were bio banked for further analysis.” |
| **Comment 11**:  Material and methods  What do the authors mean by “processed”? The authors are advised to use specific language. | Material and methods  We replaced the word "processed" in the sentence: All these strains were “analyzed” locally before shipment to Brussels by All these strains were “analyzed” locally before shipment to Brussels with "analyzed" |
| **Comment 12**:  Material and methods  The authors mentioned “statistical analysis” but do not describe which statistics they performed. | Material and methods  The data used in our study focused on the proportion of bacterial sensitivity and resistance. For this, the data received was encoded in an Excel file. They were then cleaned and checked for consistency and subsequently the proportions, were calculated using R Studio 2022.02.3 Build 492. |
| **Comment 13:**  Result:  Overall comment: The authors are advised to present their findings based on the nature of the sample collected. The authors mentioned that they collected various samples, including “blood cultures, urine, vaginal swabs, perineal swabs, and pus”. The authors are recommended to report the bacterial identification profile based on the sample type. Authors are recommended to present the antibiotic resistance profile per sample type. | Result:  We acknowledge that a bacterial identification profile based on sample type would contribute more to this study; however, our objective was to contribute to the establishment of a catalogue of bacterial strains with their resistance profile.  Thus, a summary of the antimicrobial susceptibility profiles of these species is presented in different table, (see Table 2-8). **(See line 288-316)** |
| **Comment 14:**  Result  If the authors choose to mention the inconsistency of the results observed between their local and Belgian facilities, they are advised to report those inconsistencies and discuss them. | Result-discussion  We reviewed the result section and the discussion and incorporated the various possible causes that could explain the difference between the results obtained in Goma and Brussels.  **(See line 399-406)** |
| **Comment 15:**  Result  “Bacteria from the Enterobacteriaceae family, along with those of Pseudomonas or Enterococcus genus, represent the clinically significant bacteria most frequently isolated in Goma mainly from urine, vaginal or perineal swabs.” The authors are recommended to provide references for this claim. Otherwise, if the statement is made based on the result of this study, the authors are advised to be clear and not generalize to their entire city. | Result  We have reworded the paragraph for clarity: “Bacteria from the *Enterobacteriaceae* family, along with those of *Pseudomonas* or *Enterococcus genus*, represent the most frequently isolated bacteria in our study on samples from various major hospitals in the city of Goma mainly from urine, vaginal or perineal swabs.”  **(See line 241-248)** |
| **Comment 16:**  Result  “Figure 1”: The authors are advised to add a figure caption. The authors are advised to keep “Goma” if their sampling was representative of all their city, otherwise they are advised to rephrase. | Result  We have reformulated the title as follows: “Figure 1”: Distribution of bacterial species isolated from various major hospitals in the city of Goma during the study period. **(See line 247-248)** |
| **Comment 17:**  “A summary of the antimicrobial susceptibility profiles of these species is presented in Supplementary Digital Content 1, Table 1.” It is not clear what the authors call antimicrobial susceptibility in Table 1. The Table 1 provided title is: “Prevalence of ESBL isolates, AmpC phenotype, Cephamycin impermeability, Cephalosporinase+, Carbapenemase”. Authors are recommended to provide the appropriate table for their “summary of the antimicrobial susceptibility profiles”. | We have reviewed the titles, adapted and corrected them based on the tables for greater clarity. **(See line 281-316)** |
| **Comment 18:**  “Pseudomonas aeruginosa showed a worrying trend towards multidrug resistance, with 10 (71%, 42%) producing carbapenemase (table 1).” Authors are advised to avoid using subjective language: e.g., “worrying trend”. In addition, authors need to clarify the following: “10 (71%, 42%)”: 10 what? 71% of what? 42% of what? | We have revised the text as follows for clarity:” Despite the small number of strains tested (n=13), we observe alarming resistance rates for *Pseudomonas aeruginosa* strains which showed resistance to most of the antibiotics tested, including cefepime, ciprofloxacin, levofloxacin, piperacillin/tazobactam, aztreonam, ceftazidime, imipenem. (See Table1,6) (see **line 263-268)** |
| **Comment 19:**  Discussion This section reads like a jump between several points, including lab infrastructure, transport conditions, war-related issues, environmental contamination, etc. There are no clear transitions. A major part of it sounds like an introduction or problem statement rather than an interpretation of the authors’ results. The authors are advised to reconsider their paragraphs on war, climate change, and the ICRC warning, as they are not directly tied to their results. The authors are advised to limit general background repetition that is already in the introduction.  The authors are advised to clearly restructure their discussion by briefly restating the key results of their work, explaining what they mean in their local context, and systematically comparing a comparison to regional and global data showing differences or similarities, as well as the possible reasons. The authors are advised to discuss how their findings should guide empirical therapy in their communities and how they could inform public health interventions. | We further analyzed our results and presented a more detailed discussion comparing them with other studies. In addition, we highlighted the implications of our findings for the empirical treatment of certain common infections in North Kivu, including uncomplicated cystitis, complicated urinary tract infections/pyelonephritis, and other moderate infections likely caused by enterobacteria. **(See line 386-398)** |
| **Reviewer 2: Thank you for your valuable feedback** | |

| ‘’ Sorry for the delay in my response to the review of the manuscript. The study is timely in the assessment of AMR menace in subsaharan Africa and other low-resource countries with relatively diagnostic facilities in optimal utilization clinical microbiology laboratory in patient treatment and management of cases  Title-k  Abstract- Authors presented a comprehensive, concise and detailed major finding of the study in line with objective of the study  Introduction-Authors provided epidemiological background to explained the information to back up the concept of the study, which relate factors contributing to AMR, epidemiological gaps in basic diagnostic facilities , and predisposing factors in the communities. The concept and objectives well spelt out  Methodology  The section was well designed according to the objective of study, especially adopting the WHO AWAeRA in the selection of antibiotic to be tested, which is often not used routinely in Kivu  Result  The finding were well presented in tabular format and good explanation  Discussion  The study session highlighted the challegne of implementing clinical microbiology service in LMIC, contributory factors to increasing prevalence of AMR,poor antibiotic prescription pattern. However, major gap i observed is non- comparison of local finding and abroad, thus harmonise due to lack of facilities  Conclusion and References ok ‘’ |  |
| --- | --- |
| **Reviewer 3: Thank you for your valuable feedback** | |
| **Comment 1:**  Materials and method  ESBL and carbapenemase production was detected by VITEK 2. But why do you still write that this detection was performed using phenotypic methods such as disc diffusion and chromogenic media? Which chromogenic media did you use? This part of your methodology is not clear at all. | Materials and method  We have revised the "Materials and Methods" section to include more detailed explanations of potential biases, such as the limitations of self-reported data. We believe these additions will improve understanding of the study's limitations and enhance its quality.   1. Regarding the detection of ESBL and carbapenemase production, this was done using the VITEK 2 expert system |
| **Comment 2:**  Materials and method  The described method makes no mention of the processing of the 420 isolates once they arrived at the reference laboratory in Belgium. However, these isolates were processed, and 90 were excluded. What were the exclusion criteria? How did you suspect the five Vibrio cholerae isolates? Was it based on an identification in the DRC? If so, how? These exclusion criteria must be included in your methodology. | We acknowledge the relevance of the exclusion criteria for certain bacterial strains in this study, particularly Vibrio cholerae. Included in our revised manuscript.  Regarding the exclusion criteria, isolates that were non-viable or did not grow, were misidentified, lost during transport were not included. All the bright, large, slightly flattened, yellow colonies on selective Thiosulfate Citrate Bile Sucrose agar (TCBS) after 18 to 24 hours of growth at 35 ±2 º C were suspected of being Vibrio and were also excluded. **(See line 200-204)** |
| **Comment 3:**  Materials and method  Preliminary identification of isolates (API method) was performed in the DRC and Belgium, followed by MALDI-TOF mass spectrometry analysis. Ideally, a comparison of these two identification methods and calculation of the concordance kappa coefficient should have been carried out. This would have allowed validation of the identification technique used in the DRC laboratory and identification of areas for improvement in diagnosis. | We acknowledge that Cohen's Kappa coefficient would have allowed us to validate the identification technique used in the DRC and Brussels laboratories in order to identify areas for improvement in diagnosis. However,  We were unable to calculate Cohen's Kappa coefficient because the results considered in this study were those of the reference laboratory in Brussels due to the constraints mentioned in the methodology section, notably due to a lack of reagents, resulting from supply difficulties in eastern DRC linked to insecurity.  We consider this for our future research studies |
| **Comment 4:**  Result  The figures and percentages presented in the result are incorrect. The percentage of Enterobacteriaceae is 59.4, not 59.2%. Why was 331 used as the denominator to calculate the percentage of Staphylococcus and non-fermentative bacteria? The percentage of Staphylococcus and Enterococcus is 11.2, not 11%. The sum of the bacteria families mentioned (Enterobacteriaceae, Staphylococcus or Enterococcus, Pseudomonas, and Stenotrophomonas) is 84.8%, and what about the remaining 15.2%? | We have revised the percentages presented in the result. The percentage of Enterobacteriaceae is 59.4, the percentage of *Staphylococcus* and *Enterococcus* is 11.2.  After checking, the percentage presented in the Figure appears to be correct:  *Escherichia coli* 48% (n=111), *Klebsiella pneumoniae* 13 %(n=29), *Enterococcus faecalis* 12 %(n=28), *Enterobacter cloacae* 6%(n=13), *Pseudomonas aeruginosa 6%(n=13), Citrobacter freundii 5*%(n=12), *Proteus mirabilis* 4% (n=10), Enterococcus faecium 3%(n=7), Other bacteria: *Morganella morganii*, *Citrobacter braakrii, Citrobacter Koserii, Klebsiella aerogenes, Klebsiella oxytoca, (3* %). The sum is 100 % **(see line 276)** |
| **Comment 5:**  Among the 330 isolates, 233 were considered clinically significant. What are the criteria for classifying the 233 isolates as clinically significant? What are the reasons that led you to identify 97 isolates as bacterial contaminants? 18 blood culture isolates are considered contaminants, including 5 Staphylococcus spp, 5 Bacillus spp, and 4 Acinetobacter spp. What about the remaining 4 isolates? What species are they? | The isolates considered clinically significant were the germs most frequently involved in common infections in human clinical practice, such as *Escherichia coli*, *Klebsiella pneumoniae, Enterococcus faecalis, Enterobacter cloacae, Pseudomonas aeruginosa, Citrobacter freundii, Proteus mirabilis, Enterococcus faecium.* |
| **Comment 6:**  VITEK 2 was used for AST, where are the MIC results? supplement digital content 1 should be included in the manuscript and improved by presenting the resistance profiles of all isolates. Figure 1 and Table 1 are repeated in the supplementary material. | We have revised the "Results" section by reorganizing the various tables and incorporating the MIC for improved clarity. We believe these additions will enhance understanding of the study and enrich the article. |
| **Comment 7:**  You tested 196 Enterobacteriaceae, not 175. This needs to be corrected. | We have corrected this. (**See line 269**). |

Thank you once again for your valuable feedback and suggestions. We have carefully reviewed and addressed each of your comments to enhance the clarity and quality of our manuscript. We appreciate your time and effort in providing these insights.

We look forward to your further feedback.

Sincerely,

Dr. Busha Tibasima Emmanuel
